# The effects of mitochondrial Ca^2+^ transport on intracellular Ca^2+^ waves in cardiomyocytes

**DOI:** 10.1186/1479-5876-10-S2-A67

**Published:** 2012-10-17

**Authors:** Zhenghang Zhao, Dandan Xiao, Nadezhda Fefelova, Lai-Hua Xie

**Affiliations:** 1 Department of Pharmacology, Medical School, Xi’an Jiaotong University, Xi’an 710061, China; 2 Department of Cell Biology & Molecular Medicine, UMDNJ-New Jersey Medical School, Newark NJ07103, USA

## Background

Recent studies have implicated that mitochondria play important roles in intracellular Ca^2+^ homeostasis of cardiac myocytes. The major pathways for mitochondrial Ca^2+^ transport include mitochondrial Ca^2+^ uniporter and Na^+^/Ca^2+^ exchanger, as well as mitochondrial permeability transition pore (mPTP) under certain pathophysiological conditions. However, it is still unclear if mitochondrial Ca^2+^ flux can affect the generation of Ca^2+^ waves and triggered activities in cardiomyocytes.

## Methods and results

Cytosolic Ca^2+^ (Ca_i_^2+^) was imaged in fluo-4-AM loaded ventricular myocytes isolated from mice. Spontaneous SR Ca^2+^ release and Ca^2+^ waves (CaWs) were induced in the presence of high external Ca^2+^ (Ca_o_^2+^, 4 mM). The protonophore carbonyl cyanide *p* - (trifluoromethoxy) phenylhydrazone (FCCP) reversibly raised basal Ca_i_^2+^ levels in the presence, as well as absence of Ca_o_^2+^, suggesting Ca^2+^ release from intracellular stores. Mitochondrial membrane potential (ΔΨm) was monitored by TMRM fluorescence. FCCP at 0.01- 0.1 µM, which partially depolarized ΔΨm, increased the frequency and amplitude of CaWs in a dose-dependent manner. Simultaneous recording of cell membrane potentials showed the augmentation of delayed after depolarization amplitudes and frequencies, and induction of triggered action potentials. On the contrary, FCCP at higher concentrations (>0.5 µM), which completely dissipated ΔΨm, eliminated CaWs while the basal Ca_i_^2+^ remained high. The cease of CaWs was most likely due to the reduction of SR Ca^2+^ content as evaluated by rapid exposure to10 mM caffeine. Blocking sarcolemmal Na^+^-Ca^2+^ exchanger by substituting Na^+^ with Li^+^ in the perfusant further elevated basal Ca_i_^2+^ and restored CaWs. The effect of FCCP on CaWs was mimicked by antimycin A (an electron transport chain inhibitor disrupting ΔΨm) or Ru360 (a mitochondrial Ca^2+^ uniporter inhibitor), but not by oligomycin (an ATP synthase inhibitor) or iodoacetic acid (a glycolytic inhibitor), excluding the contribution of intracellular ATP levels. The effects of FCCP on CaWs were counteracted by the mitochondrial permeability transition pore blocker cyclosporine A, or the mitochondrial Ca^2+^ uniporter activator kaempferol.

## Conclusions

Mitochondrial Ca^2+^ release and uptake control plasma Ca^2+^ levels and plays an important role in regulation of intracellular CaWs and arrhythmogenesis.

